# Pharmacodynamic Effect of Different Dosage Regimes of Oseltamivir in Severe Influenza Patients Requiring Mechanical Ventilation: A Multicentre Randomised Controlled Trial

**DOI:** 10.1111/irv.70109

**Published:** 2025-05-19

**Authors:** Wai‐Tat Wong, Gordon Choi, Xiansong Wang, William Ka Kei Wu, Ge Lin, Martin Chi Wai Chan, King Chung Kenny Chan, Philip Koon Ngai Lam, David Shu Cheong Hui, Matthew T. V. Chan

**Affiliations:** ^1^ Department of Anaesthesia and Intensive Care The Chinese University of Hong Kong Hong Kong SAR China; ^2^ Department of Anaesthesia and Intensive Care Prince of Wales Hospital Hong Kong SAR China; ^3^ School of Biomedical Sciences The Chinese University of Hong Kong Hong Kong SAR China; ^4^ Department of Microbiology The Chinese University of Hong Kong Hong Kong SAR China; ^5^ Department of Intensive Care Tuen Mun Hospital Hong Kong SAR China; ^6^ Department of Intensive Care North District Hospital Hong Kong SAR China; ^7^ Department of Medicine and Therapeutics The Chinese University of Hong Kong Hong Kong SAR China

**Keywords:** mechanical ventilation, oseltamivir, severe influenza

## Abstract

**Background and Objectives:**

This randomised controlled trial evaluated whether higher doses of oseltamivir would improve virological and clinical outcomes in severe influenza patients requiring invasive mechanical ventilation.

**Methods:**

Forty intubated adult patients with severe influenza A or B from four intensive care units in Hong Kong were enrolled and randomised to receive either a double dose (300 mg/day) or a triple dose (450 mg/day) of oseltamivir for 10 days. Baseline data were collected, and outcomes were assessed daily using SOFA and Murray scores. Viral RNA was quantified from nasopharyngeal and tracheal aspirates. The primary outcome was the viral clearance rate after 5 days of treatment; secondary outcomes included 28‐day and hospital mortality rates, changes in viral load, and serial SOFA and Murray scores.

**Results:**

Viral clearance rates after 5 days of treatment were low and similar between the double (3/20, 15%) and triple‐dose groups (2/20, 10%). No significant differences were observed in 28‐day mortality, hospital mortality, ICU length of stay or duration of mechanical ventilation between the double and triple‐dose groups. However, patients receiving triple doses exhibited a faster decline in influenza A viral load but had a longer hospital length of stay.

**Conclusions:**

Triple doses of oseltamivir did not significantly improve virological or clinical outcomes compared with double doses in severe influenza.

## 1 Introduction

Acute respiratory failure is a serious complication of severe influenza and is associated with a high mortality rate of > 10% [1, 2]. Oseltamivir is a competitive antagonist of neuraminidase enzymes that prevents the release of new virions from cells infected by influenza A or B virus. In a meta‐analysis of 29,234 patients hospitalised with influenza A H1N1pdm09, the standard dose (150 mg/day) of oseltamivir and other neuraminidase inhibitors administered within 2 days of symptom onset decreased mortality [3]. In animal experiments, higher doses of oseltamivir (equivalent to 300 mg/day) improved survival in mice [4] and ferrets [5] infected with the H5N1 influenza virus. Based on these preclinical studies, the World Health Organization (WHO) recommended a double dose of oseltamivir (300 mg/day) for critically ill patients infected with the pandemic influenza A (H1N1) 2009 virus in 2010 [6]. Subsequently, the Infectious Disease Society of America (IDSA) advised against the routine use of higher doses of oseltamivir for seasonal influenza in 2018 [7]. The guidelines for the treatment of severe diseases caused by zoonotic novel influenza A published by the Centres for Disease Control and Prevention (CDC) indicate that the dosage remains uncertain [8]. Most recently, in 2024, WHO recommended a standard dose of oseltamivir (150 mg/day) for patients with severe seasonal influenza or novel influenza A infections [9]. Thus far, there are two retrospective studies evaluating the clinical outcomes of ICU influenza patients receiving double doses of oseltamivir (300 mg/day) [10, 11]. Dose‐dependent improvements in both virological and clinical outcomes with triple doses of oseltamivir (450 mg/day) have yet to be evaluated by a randomised controlled trial.

In this trial, we hypothesised that higher doses of oseltamivir would more effectively suppress viral replication and improve organ functions. We conducted a randomised controlled trial to compare the virological and clinical outcomes of critically ill patients with influenza who received double (300 mg/day) or triple doses (450 mg/day) of oseltamivir for 10 days.

## 2 Methods

This was a prospective, randomised, double‐blinded trial. Eligible patients were recruited from intensive care units (ICU) of four public hospitals in Hong Kong. The rationale and design of the trial were reported to the Centre for Clinical Research and Biostatistics – Clinical Trials Registry (CCRBCTR) [ChiCTR‐IOR‐15006823]. The trial was approved by the Joint Chinese University of Hong Kong New Territories East Cluster Clinical Research Ethics Committee (2014.679.T), and written informed consents were obtained from patients or their next‐of‐kin.

We studied adult patients aged 18 years or older suffering from laboratory‐confirmed influenza A or B who required invasive mechanical ventilation of the lungs. We excluded patients who received oseltamivir for more than 2 days prior to ICU admission, patients who were allergic to oseltamivir, pregnant patients, and patients who required renal replacement therapy at the time of admission.

Following enrolment, patients were randomly assigned to receive either a double dose (300 mg/day) or a triple dose (450 mg/day) of oseltamivir for a minimum duration of 10 days in a 1:1 ratio, owing to the anticipated prolonged virus shedding in critically ill patients [12]. Further extension of treatment beyond 10 days, subject to the attending physicians' judgment, was permitted based on clinical progress. The allocated dosages were not permitted to be altered unless renal function deteriorated to the stage necessitating renal replacement therapy and exclusion from the study. Computer‐generated randomisation codes were prepared and concealed in opaque envelopes by the research staff for consecutive patients enrolled in all participating ICUs. The trial drug was prepared by the hospital pharmacy. Oseltamivir powder extracted from the drug capsule was dissolved in 20 mL of sterile water at room temperature and was administered through a nasogastric feeding tube, followed by 20 mL water flush, as previously described in the study evaluating the pharmacokinetics of oseltamivir in critically ill patients [13]. The healthcare team managing the enrolled patients were aware of the treatment allocation, but both the research staff evaluated the outcomes, and patients were blinded to the treatment allocation.

We collected baseline data to evaluate co‐morbidity and disease severity using the Charlson comorbidity index and the Acute Physiology and Chronic Health Evaluation (APACHE) II scores, respectively [14, 15]. Daily organ functions, including the respiratory, cardiovascular, neurological, renal, haematological and hepatobiliary systems, were recorded using the Sequential Organ Function Assessment (SOFA) score. Changes in lung function, including oxygenation, radiological appearance and lung mechanics over time, were evaluated daily using the Murray score for acute lung injury [16]. In addition, we recorded the length of stay in the hospital and ICU, as well as the duration of invasive mechanical ventilatory support.

We collected nasopharyngeal and tracheal aspirate for quantification of viral RNA at baseline and then daily until day 10 of ICU admission. Influenza A and B viral RNA was measured by quantitative reverse transcription PCR targeting the M‐gene (SuperScript III Platinum One‐Step qRT‐PCR Kit w/ROX; Invitrogen, Carlsbad, CA), as previously described [12, 17]. Viral clearance was defined as the day with no viral RNA detection from tracheal aspirate in patients receiving invasive mechanical ventilation or nasopharyngeal aspirate after tracheal extubation. Similarly, viral load from nasopharyngeal aspirate instead of tracheal aspirate was used after tracheal extubation for longitudinal viral load assessment. The primary outcome was the rate of viral clearance after 5 days of oseltamivir treatment, as noted in a previous oseltamivir dose comparison study [18, 19]. Secondary outcomes were 28‐day mortality after randomisation, hospital mortality, changes in viral loads, SOFA and Murray score during the first 10 days after randomisation.

### 2.1 Sample Size Calculation

The sample size was calculated based on a change in the viral clearance rate from 35% to 80%, as previously reported in critically ill patients receiving a triple dose (450 mg/day) [18] in critically and noncritically ill patients taking a double dose (300 mg/day) of oseltamivir [18, 19]. A total of 20 patients per group was required to detect the difference with 80% power, assuming a one‐sided α of 0.05 and a 10% noninclusion rate.

### 2.2 Data Analysis

All analyses were performed according to the intention‐to‐treat principle. Categorical data were analysed by χ^2^ or Fisher exact test, as appropriate. Continuous variables were assessed for normality using the Shapiro–Wilk test, and nonparametric data were analysed by the Mann–Whitney *U* test. The primary and secondary outcomes were compared between the two doses using logistic regression. Analyses were adjusted based on clinically important factors, including gender, Charlson comorbidity score, APACHE II score, type of influenza infection and bacterial coinfection. Differences in length of ICU stay, hospital stay and duration of mechanical ventilation between two doses of oseltamivir were analysed by multiple linear regression with the same set of covariate adjustments. Longitudinal data, including daily viral load, SOFA and Murray scores of patients who received two doses, were analysed using a linear mixed‐effect model with Bayesian Joint Modelling (imputation of incomplete data) [20]. Analyses were performed using SPSS Statistics for Windows (version 24, IBM Corp, Chicago, IL, USA) and R programme (Joint Analysis and Imputation of Incomplete data package).

## 3 Results

**FIGURE 1:**
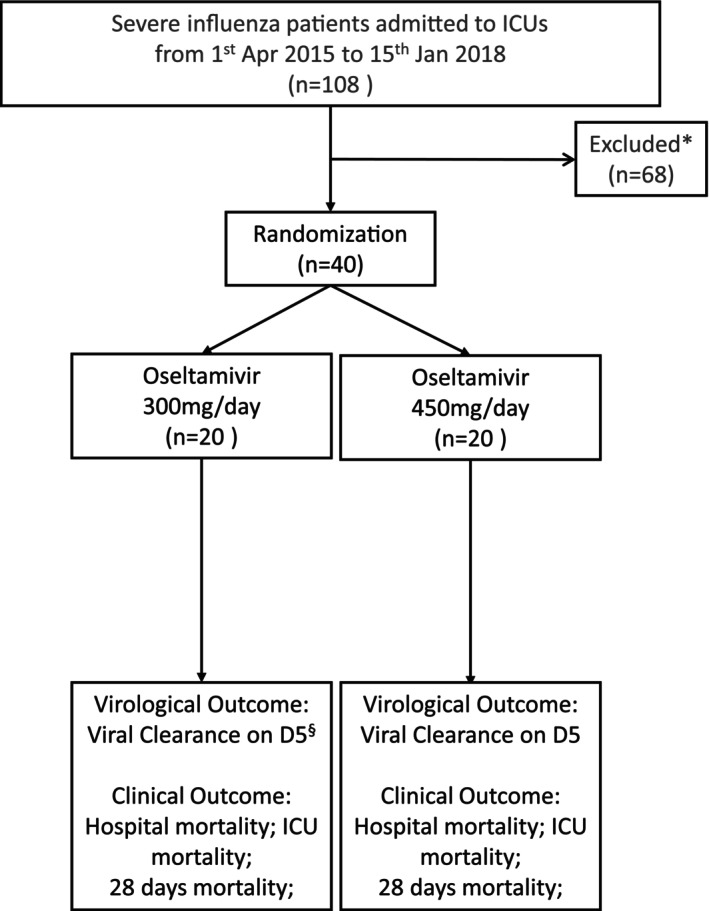
CONSORT flow diagram of patient enrolment. “*” represents 26 patients did not require invasive ventilator support; 29 patients required renal replacement therapy; 5 patients died shortly after ICU admission/adopted limitation of support; 4 patients had delayed screening and consent (> 48 h after ICU admission); 3 refused to participate; 1 patient was pregnant. “§” represents 2 patients who had the missing virological outcome due to early discharge and early death, and viral clearance and viral persistence were assumed based on the clinical ground, respectively (*N* = 18).

A total of 108 consecutive patients admitted to the ICU with severe influenza from the four participating hospitals were screened between 1 April 2015 and 15 January 2018. Sixty‐eight patients were excluded [26 patients did not require ventilator support; 29 patients required renal replacement therapy; 5 patients died or adopted limitation of support shortly after ICU admission, making the enrolment and randomisation not feasible; 4 patients had delayed screening and consent (> 48 h after ICU admission); 3 refused to participate; 1 patient was pregnant]. Finally, 40 patients were randomised to double dose (300 mg/day, *n* = 20) or triple dose (450 mg/day, *n* = 20) of oseltamivir and two randomised patients from the double dose group were lost for evaluation of primary outcome, viral clearance after 5 days of oseltamivir treatment, due to early discharge from ICU and early death before the evaluation (Figure 1).

Table 1 shows the demographic characteristics of patients at ICU admission. Patients randomised to either dose of oseltamivir had similar severity of illness and extent of comorbidity. Patients receiving triple doses had higher oseltamivir carboxylate trough concentration and creatinine clearance compared to patients receiving double doses. However, both parameters were not statistically significant. Outcomes are summarised in Table 2. Imputing the result of viral clearance to the early discharged patient and viral persistence to the early died patient, the rate of viral clearance on day 5 after randomisation was low (3/20 (15%) in patients receiving oseltamivir 300 mg/day and 2/20 (10%) in patients receiving 450 mg/day) and similar in patients receiving double or triple doses (*p* = 0.637) using the intention‐to‐treat principle in the analysis. The Hosmer–Lemeshow goodness‐of‐fit test yielded a *χ* ^2^ value of 16.146 (*p* = 0.040), suggesting a moderate model fit. Similar results were found with the analysis using the per‐protocol principle (Table S1). The hospital and 28‐day mortality rates were not significantly different between the two groups of patients receiving double (300 mg/day) or triple doses of oseltamivir (450 mg/day) (*p* = 0.125 and *p* = 0.098). The duration of mechanical ventilation and ICU length of stay were similar for patients receiving two different doses of oseltamivir, but the hospital length of stay was longer for patients receiving triple doses of oseltamivir (450 mg/day).

**TABLE 1:** Characteristics of the patients randomised to control and intervention groups.

	Double dose (oseltamivir 300 mg/day), *N* = 20	Triple dose (oseltamivir 450 mg/day), *N* = 20	*p* *
Gender (male %)	12/20 (60%)	9/20 (45%)	0.342
Age (median, IQR)	60.5 (48.5–66.8)	59.0 (48.0–63.8)	0.602
Body weight (kg)	62.2 (55.9–73.0)	59.0 (52.0–65.0)	0.527
Body mass index (BMI) (median, IQR)	24.9 (20.8–27.8)	22.1 (19.8–26.7)	0.862
Influenza A (%)	19/20 (95%)	16/20 (80%)	0.151
Bacterial coinfection (%)	7 (35%)	9 (45%)	0.519
APACHE II score (median, IQR)	18.5 (15.3–23.3)	18.5 (16.3–23.5)	0.925
Charlson's score (median, IQR)	3 (1–4)	2 (1–3)	0.277
Oseltamivir carboxylate trough concentration (μg/L) (median, IQR)	782.4 (604.3–1011.6)	926.1 (296.2–1726.0)	0.839
Oseltamivir carboxylate trough concentration (μg/L/kg) (median, IQR)	0.0129 (0.0069–0.0160)	0.0142 (0.0063–0.0266)	0.839
Measured creatinine clearance (mL/min) (median, IQR)	60.7 (28–5‐88.4)	76.4 (41.1–119.3)	0.752

* Univariate analysis by chi‐square or Mann–Whitney *U* test.

**TABLE 2:** Primary and secondary outcomes of the patients randomised to control and intervention groups.

	**Double dose (oseltamivir 300/day), *n* = 20**	**Triple dose (oseltamivir 450 mg/day), *n* = 20**	**Adjusted *R*** ^**2**^ **(95% confidence interval)** ^**a**^	***p***
Intubation duration (h) (median, IQR)	158.0 (71.5–400.9)	213.7 (131.2–340.8)	459.784 (−280.152–1199.720)	0.215
ICU length of stay (days) (median, IQR)	9.0 (4.2–22.6)	12.3 (9.1–17.2)	26.273 (−13.247–65.793)	0.186
Hospital length of stay (days) (median, IQR)	12.9 (8.1–34.9)	22.6 (17.5–34.5)	56.896 (14.926–98.866)	0.009
			**Odd ratios (95% confidence interval)** ^**b**^	***p***
Day 5 viral clearance	3/20 (15%)	2/20 (10%)	2.038 (0.069–60.200)	0.680
28 days mortality	5/20 (25%)	1/20 (5.0%)	15.111 (0.272–839.321)	0.185
Hospital mortality	6/20 (30%)	1/20 (5.0%)	21.797 (0.349–1361.289)	0.144

^a^ Multivariate analysis by multiple linear regression: adjusted for covariate: gender, APACHE II score, Charlson's score, influenza type and bacterial coinfection.

^b^ Multivariate analysis by logistic regression: adjusted for covariate: gender, APACHE II score, Charlson's score, influenza type and bacterial coinfection.

**FIGURE 2:**
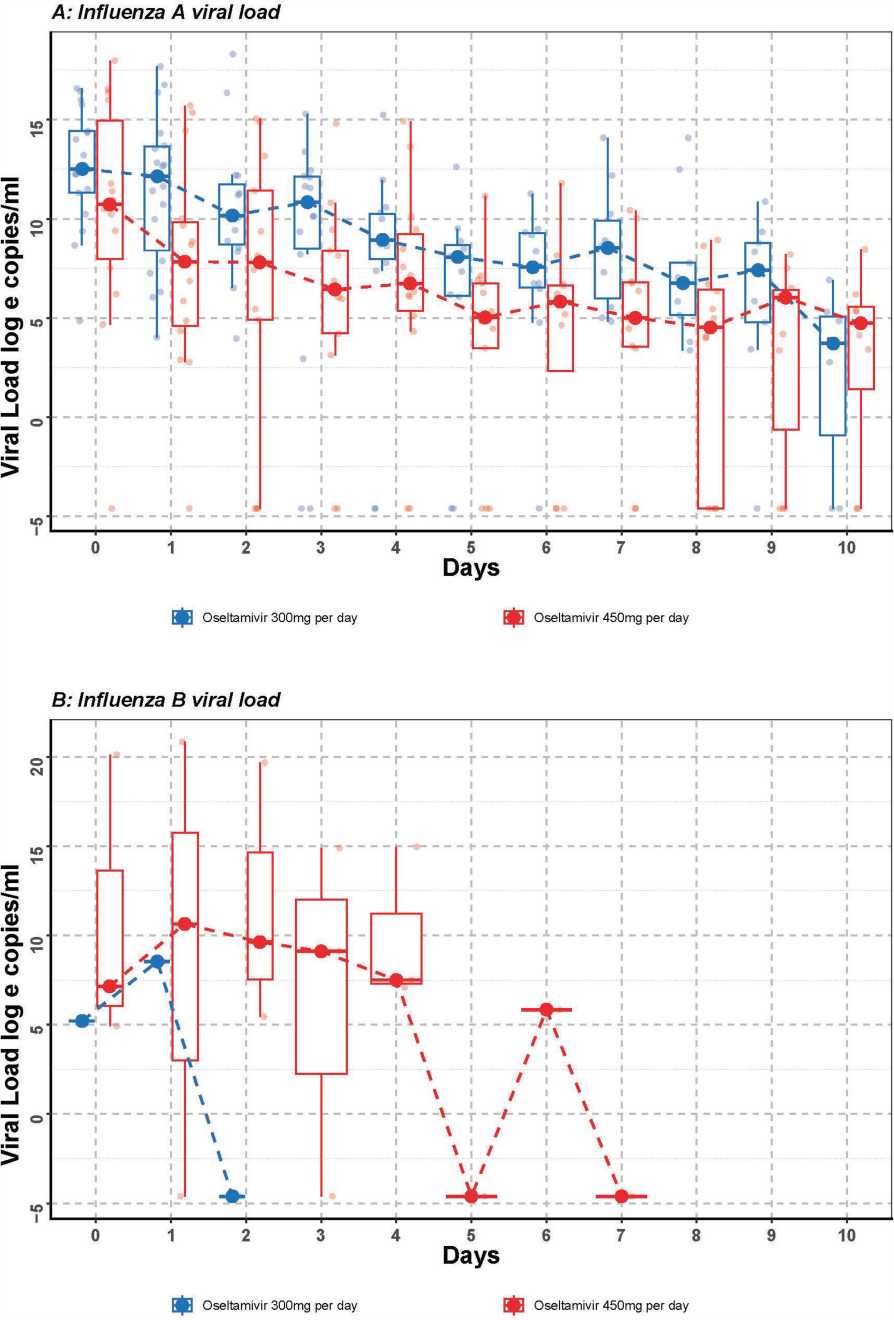
(A, B) Influenza A and B viral load. Viral load from day 0 to day 10 of recruited Influenza A patients (*N* = 35) and Influenza B patients (*N* = 5). The longitudinal viral load was analysed by a generalised mixed‐effect model adjusting for gender, Acute Physiologic Assessment and Chronic Health Evaluation (APACHE) II score, Charlson's score and bacterial coinfection status (Influenza A patients: *p* = 0.048 and Influenza B patients: insufficient data to analyse).

**FIGURE 3:**
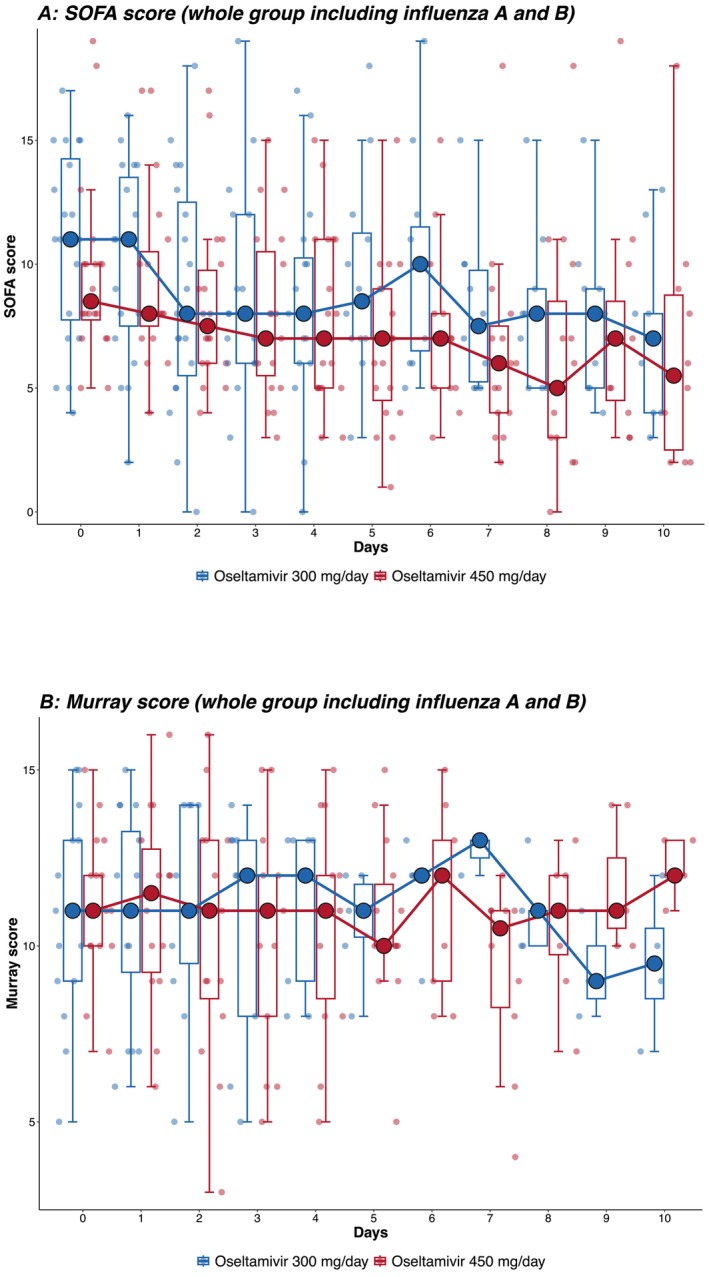
(A, B) SOFA and Murray scores. SOFA and Murray scores from day 0 to day 10 of all influenza patients (Influenza A and Influenza B, *N* = 40). The longitudinal SOFA score was analysed by a generalised mixed‐effect model adjusting for gender, Acute Physiologic Assessment and Chronic Health Evaluation (APACHE) II score, Charlson's score, influenza type and bacterial coinfection status (SOFA score: *p* = 0.144, Murray score: *p* = 0.624).

The rate of decrease in viral load from tracheal aspirate in patients infected by influenza A was faster in patients receiving triple doses (450 mg/day) of oseltamivir compared with double doses (300 mg/day), adjusted for disease severity at ICU admission and comorbidity index (*β* = −3.19, 95% CI: −6.19 to −0.23, *p* = 0.043) (Figure 2A). There was insufficient data to analyse the effect of dose on viral load among patients infected with influenza B (Figure 2B). There were similar rates of decrease in daily SOFA (*β* = −1.73, 95% CI: −4.06 to 0.66, *p* = 0.144; Figure 3A) and Murray scores (*β* = −0.04, 95% CI: −0.22 to 0.13, *p* = 0.624; Figure 3B) in patients between groups. No enrolled patients in either group required stopping and dosage adjustments due to adverse effects.

## 4 Discussion

This study examined the effects of double (300 mg/day) or triple doses of oseltamivir (450 mg/day) on the clinical and virological outcomes of severe influenza patients who require invasive mechanical ventilation in ICUs. The study found that viral clearance rates were low on the fifth day of treatment, and there was no difference between patients receiving double or triple doses of oseltamivir. There was also no significant difference in mortality, ICU length of stay and duration of invasive mechanical ventilation between the two groups. However, patients receiving triple doses of oseltamivir showed a trend of faster viral load decline and organ function improvement, as measured by the daily SOFA and Murray scores.

Studies have shown that prescribing oseltamivir up to six times the standard dose (900 mg/day) is safe [21]. Randomised controlled trials have attempted to compare the effectiveness of higher doses of oseltamivir with standard doses (150 mg/day) in hospitalised patients with influenza [19, 22]. Although there was a trend towards more rapid decline in viral load of influenza B in hospitalized influenza patients receiving a double dose of oseltamivir (300 mg/day) [22], there was no difference in viral clearance rate after 5 days of oseltamivir in two previous studies conducted in Asia. These two studies recruited adult and paediatric patients requiring hospitalisation and sometimes oxygen supplementation; the proportion of critically ill patients was less than 10% [19, 22]. Therefore, based on this evidence, it is difficult to justify prescribing higher than the standard dose of oseltamivir for critically ill patients requiring invasive mechanical ventilation in ICUs.

Subsequently, two retrospective studies conducted in ICUs found no evidence of improved clinical outcomes in patients receiving double doses of oseltamivir (300 mg/day) compared to those receiving standard doses of oseltamivir (150 mg/day) [10, 11]. The Trial Comparing High Versus Standard Dose Oseltamivir in Severe Influenza Infection in ICU (ROSII) investigators reported the preliminary findings of a higher viral clearance rate in critically ill influenza patients receiving 5 days of triple doses of oseltamivir compared to standard doses (78% vs 11%) in the 53rd Interscience Conference on Antimicrobial Agents and Chemotherapy. However, the difference was from the analysis of only nine patients randomised to each arm, and the ROSII study was subsequently terminated due to the difficulty in recruiting adequate patients [18].

A previous pharmacokinetic study discovered that serum concentration of oseltamivir carboxylate was 2000‐ to 4000‐fold higher than the documented 50% maximal inhibitory concentration of oseltamivir to influenza virus in critically ill patients receiving a standard dose of oseltamivir [13]. The serum concentration of oseltamivir carboxylate in patients from both arms of our study was higher than in the previously mentioned study. Based on our findings of similar virological and clinical outcomes in patients receiving double (300 mg/day) or triple doses of oseltamivir (450 mg/day) and on previous studies comparing the treatment effectiveness between double (300 mg/day) and standard doses (150 mg/day), critically ill patients who require invasive ventilator support should not be prescribed higher than the standard dose of oseltamivir (150 mg/day), which is the current recommended dosage for hospitalised influenza patients [9]. More randomised controlled trials on the timely use of antiviral combination (oseltamivir plus baloxavir) versus oseltamivir alone on clinical outcome are needed in managing patients hospitalised with severe influenza [23].

This is the first randomised controlled trial to explore the potential differences in virological and clinical outcomes of critically ill patients receiving triple doses of oseltamivir, compared with those receiving double doses. There are several limitations of this study. (1) The small sample size is likely insufficient to detect differences in mortality and other clinical outcomes between the two groups. The only significant difference in clinical outcome, the longer hospital stay associated with triple doses of oseltamivir, could be related to factors other than oseltamivir dosage but not adjusted in the analysis. No enrolled patient required drug cessation or adjustment due to adverse effects. (2) Regarding the viral negativity after 5 days of treatment, the primary outcome of the study, there was no viral load assessments conducted from either tracheal or nasopharyngeal aspirate for two patients in the double dose (300 mg/day) group. Viral clearance was assumed for the patient who improved and was discharged early, while viral persistence was presumed for the patient who died early based on the rapid clinical improvement and subsequent deterioration due to severe influenza, to uphold the intention‐to‐treat principle for managing missing values. The same insignificant difference was also confirmed in the per‐protocol analysis by excluding these two patients from the primary outcome analysis (Table S1). (3) The finding of a significantly faster decline in longitudinal influenza A viral load did not encompass all enrolled patients, which may represent a chance finding. (4) The reference starting point for this study was ICU admission rather than the onset of symptoms. While initiating oseltamivir within 48 h of symptom onset is recommended [9], we could not assess the impact of prolonged symptoms prior to ICU admission, which could affect the effectiveness of oseltamivir. (5) The difference in serum concentration of oseltamivir carboxylate between patients receiving double and triple doses was not statistically significant. Drug compliance should be ensured in the participating ICUs as all double or triple doses of oseltamivir were prepared by the pharmacy and administered to the enrolled patients via gastric tube by the nurses. The allocated oseltamivir regimens should have been fully delivered to the enrolled patients. The lack of difference in oseltamivir carboxylate concentration may relate to varying drug absorption resulting from gastrointestinal malfunction, conversion from oseltamivir phosphate due to hepatic failure, and drug elimination owing to renal failure, as indicated by the differing creatinine clearances between the two patient groups [24]. The small sample size hindered the identification of differences in organ function, as evidenced by the nonsignificant statistical difference in creatinine clearance. Only patients requiring renal replacement therapy were excluded, while those with renal failure not necessitating such therapy were prescribed the allocated doses of oseltamivir. (6) Details regarding side effect profiles were not collected in our study. However, gastrointestinal dysfunction and mild cognitive impairment are commonly reported side effects of oseltamivir. Nevertheless, these symptoms may be attributed to mechanisms other than the administration of oseltamivir in ICU patients, who frequently receive concurrent broad‐spectrum antibiotics and experience multi‐organ failure, including diarrhoea, gastroparesis and delirium. All recruited patients did not require cessation or adjustment of oseltamivir due to adverse effects during the study period.

## 5 Conclusion

Triple doses of oseltamivir (450 mg/day) did not more effectively suppress the viral replication or improve organ function of critically ill influenza patients requiring invasive mechanical ventilation than double doses (300 mg/day). Triple doses of oseltamivir (450 mg/day) are not indicated for critically ill influenza patients.

## Author Contributions

**Wai‐Tat WONG:** conceptualization, methodology, writing – original draft, writing – review and editing, project administration, resources, formal analysis, validation, investigation, visualization, funding acquisition, data curation, software. **Gordon Choi:** conceptualization, investigation, funding acquisition, writing – original draft, methodology, validation, visualization, writing – review and editing, formal analysis, project administration, data curation, resources, supervision. **Xiansong Wang:** methodology, software, formal analysis, validation, writing – review and editing. **William Ka Kei Wu:** writing – review and editing, funding acquisition, investigation, methodology, formal analysis, software. **Ge Lin:** formal analysis, data curation, supervision. **Martin Chi Wai Chan:** investigation, methodology, formal analysis, writing – review and editing, data curation, methodology, writing – review and editing. **King Chung Kenny Chan:** investigation, methodology, formal analysis, writing – review and editing, data curation, methodology, writing – review and editing. **Philip Koon Ngai Lam:** methodology, data curation, writing – review and editing. **David Shu Cheong Hui:** conceptualization, supervision, writing – review and editing, funding acquisition, investigation, methodology, formal analysis. **Matthew T. V. Chan:** conceptualization, methodology, investigation, funding acquisition, writing – original draft, writing – review and editing, supervision, data curation, formal analysis, validation, resources.

## Ethics Statement

The rationale and design of the trial were reported at the Centre for Clinical Research and Biostatistics – Clinical Trials Registry (CCRBCTR)[ChiCTR‐IOR‐15006823]. The trial was approved by the Joint Chinese University of Hong Kong New Territories East Cluster Clinical Research Ethics Committee (2014.679.T), and written informed consents were obtained from patients or their next‐of‐kin.

## Conflicts of Interest

The authors declare no conflicts of interest.

### Peer Review

The peer review history for this article is available at https://www.webofscience.com/api/gateway/wos/peer‐review/10.1111/irv.70109.

## Supporting information


**Table S1.** Primary and secondary outcomes of the patients randomised to control and intervention groups (per protocol analysis)

**Table S2.** Supporting Information

## Data Availability

The data that support the findings of this study are available from the corresponding author upon reasonable request.
