# Supplementary material for: Pharmacodynamic Effect of Different Dosage Regimes of Oseltamivir in Severe Influenza Patients Requiring Mechanical Ventilation: A Multicentre Randomised Controlled Trial
Source: Influenza Other Respir Viruses. 2025 May 19;19(5):e70109. doi: 10.1111/irv.70109 (PMC12086322; doi:10.1111/irv.70109)
Supplement: Supplementary file 1 — Table S1. Primary and secondary outcomes of the patients randomised to control and intervention groups (per protocol analysis) [file IRV-19-e70109-s002.docx]

Supplementary Table 1: Primary and secondary outcomes of the patients randomised to control and intervention groups (per protocol analysis)

|  | Double dose (Oseltamivir 300/day)  (n=18) | Triple dose  (Oseltamivir 450 mg/day)  (n=20) | Adjusted R^2^ (95% Confidence Interval)^†^ | *p*-value |
| --- | --- | --- | --- | --- |
| Intubation duration (hours)  (median, IQR) | 158.0  (109.1-352.6) | 227.0  (116.3-449.9) | 485.809  (-302.856 - 1274.473) | 0.219 |
| ICU length of stay (days) (median, IQR) | 9.0  (4.5-21.7) | 12.7  (6.8-19.6) | 26.463  (-14.881 – 67.807) | 0.202 |
| Hospital length of stay (days) (median, IQR) | 17.3  (8.4-32.6) | 23.4  (16.6-35.5) | 58.335  (14.067 – 102.594) | 0.011 |
|  |  |  | **Odd ratios (95% Confidence Interval)** **^‡^** | ***p*-value** |
| Day 5 viral clearance | 2/18 (11.1%)^§^ | 2/20 (10%) | 0.117  (0.001-13.248) | 0.374 |
| 28 days mortality | 5/18 (27.8%) | 1/20 (5.0%) | 17.169  (0.415-710.271) | 0.134 |
| Hospital Mortality | 6/18 (33.3%) | 1/20 (5.0%) | 14.020  (0.480-409.398) | 0.125 |
|  |  |  |  |  |

*^†^Multivariate analysis by multiple linear regression: Adjusted for covariate: Gender, APACHE II score, Charlson’s score, Influenza type and bacterial coinfection.*

*^‡^Multivariate analysis by logistic regression: Adjusted for covariate: Gender, APACHE II score, Charlson’s score, Influenza type and bacterial coinfection.*

*^§^Two patients were lost for evaluation due to early discharge from ICU and early death before evaluation (N=18).*
