# Supplementary material for: Pharmacodynamic Effect of Different Dosage Regimes of Oseltamivir in Severe Influenza Patients Requiring Mechanical Ventilation: A Multicentre Randomised Controlled Trial
Source: Influenza Other Respir Viruses. 2025 May 19;19(5):e70109. doi: 10.1111/irv.70109 (PMC12086322; doi:10.1111/irv.70109)
Supplement: Supplementary file 2 — Table S2. Supporting Information [file IRV-19-e70109-s001.pdf]

Study number	Randomisation group	Gender	Age	BMI	Influenza type	Secondary bacterial infection	APACHEII score	Charlson's score	Oseltamivir carboxylate trough concentration (µg/L)	Measured Creatinine Clearance (ml/min)	Day 5 Viral Clearance	28 days mortality	Hospital mortality	Intubation duration (hours)	ICU LOS (Days)	Hospital LOS (Days)
1	Control group (300mg/day)	Female	45	30.17	A	No bacterial infection	17	2	0.006	98.53	Persistent viral activity	Alive	Alive	170.58	14.4	47.52
2	Intervention group (450mg/day)	Male	82	18.29	A	Bacterial infection	25	4	0.04558	39.01	Persistent viral activity	Alive	Alive	293	13.93	22.35
3	Intervention group (450mg/day)	Male	61	23.88	B	No bacterial infection	28	4	0.01425	166.12	Persistent viral activity	Dead	Dead	120.58	5.52	15.25
4	Control group (300mg/day)	Male	70		A	No bacterial infection	14	5	0.02316	65.45	Viral clearance	Alive	Alive	42.25	3.36	11.36
5	Control group (300mg/day)	Male	58	25.8	A	Bacterial infection	15	2	0.01206	88.39	Persistent viral activity	Alive	Alive	241.67	10.34	20.34
6	Intervention group (450mg/day)	Female	34	21.05	A	No bacterial infection	19	0	0.00389	146.46	Persistent viral activity	Alive	Alive	356.67	16.38	24.39
7	Control group (300mg/day)	Male	57	26.21	A	No bacterial infection	19	5	0.0159	60.66	Persistent viral activity	Alive	Alive	68.08	4.47	8.48
8	Control group (300mg/day)	Male	71		A	No bacterial infection	19	3			Persistent viral activity	Dead	Dead	262.3	10.33	10.35
9	Intervention group (450mg/day)	Female	59	21.56	A	No bacterial infection	22	2	0.01798	87.3	Persistent viral activity	Alive	Alive	87.47	15.08	22.09
10	Control group (300mg/day)	Female	26	32.02	A	No bacterial infection	15	0	0.00962	59.72	Persistent viral activity	Alive	Alive	474.17	84.43	31.44
11	Control group (300mg/day)	Female	28		A	No bacterial infection	26	0			Persistent viral activity *	Dead	Dead	18.25	0.43	0.43
12	Intervention group (450mg/day)	Female	64	14.63	B	Bacterial infection	24	4	0.04702	47.37	Viral clearance	Alive	Alive	881	38.3	53.39
13	Control group (300mg/day)	Male	42	27.76	A	Bacterial infection	21	0	0.00693	67.23	Persistent viral activity	Alive	Alive	224.5	13.24	14.41
14	Intervention group (450mg/day)	Male	58	18.73	A	No bacterial infection	19	1	0.00388	59.08	Persistent viral activity	Alive	Alive	114.83	6.24	20.25
15	Intervention group (450mg/day)	Male	54		A	No bacterial infection	11	3	0.04591	32.08	Persistent viral activity	Alive	Alive	229	10.48	18.48
16	Intervention group (450mg/day)	Female	40		A	Bacterial infection	16	2	0.00879	82.26	Persistent viral activity	Alive	Alive	203	15.79	22.79
17	Intervention group (450mg/day)	Male	64	22.76	A	No bacterial infection	16	3	0.04084	33.07	Persistent viral activity	Alive	Alive	19	3.87	35.94
18	Intervention group (450mg/day)	Female	28	27.99	A	No bacterial infection	18	0	0.00359	219.51	Persistent viral activity	Alive	Alive	193	10.81	14.97
19	Control group (300mg/day)	Male	77	19.82	A	No bacterial infection	31	3	0.0194	26.78	Persistent viral activity	Dead	Dead	118.25	4.17	4.75
20	Control group (300mg/day)	Female	62	26.67	A	No bacterial infection	17	2			Persistent viral activity	Alive	Alive	425.08	23.05	36.05
21	Control group (300mg/day)	Male	86	20.2	A	Bacterial infection	37	4	0.00017	28.49	Viral clearance	Dead	Dead	124	4.53	4.84
22	Intervention group (450mg/day)	Female	68		A	No bacterial infection	18	3		2.87	Viral clearance	Alive	Alive	211.42	12.47	34.47
23	Control group (300mg/day)	Female	63	20.75	B	No bacterial infection	21	4	0.01602	78.38	Persistent viral activity	Alive	Alive	81.83	4.8	29.95
24	Intervention group (450mg/day)	Female	63	27.39	B	Bacterial infection	24	2	0.00357	57.89	Persistent viral activity	Alive	Alive	108	-4.73	17.29
25	Intervention group (450mg/day)	Male	59	19.62	B	Bacterial infection	25	4	0.00522	62.75	Persistent viral activity	Alive	Alive	192	8.65	27.78
26	Control group (300mg/day)	Female	67	22.39	A	No bacterial infection	24	4			Persistent viral activity	Alive	Alive	63.75	2.01	8.01
27	Intervention group (450mg/day)	Female	62	20.16	A	Bacterial infection	18	3	0.0228	88.51	Persistent viral activity	Alive	Alive	482.75	20.33	34.38
29	Intervention group (450mg/day)	Female	57	28.39	A	Bacterial infection	17	2	0.00747	177.57	Persistent viral activity	Alive	Alive	276.25	11.35	16.35
30	Intervention group (450mg/day)	Male	65	23.43	A	Bacterial infection	19	2	0.0174	72.02	Persistent viral activity	Alive	Alive	163.07	17.43	32.46
31	Control group (300mg/day)	Female	56		A	Bacterial infection	13	2	0.01375	29.21	Persistent viral activity	Alive	Alive	824.75	34.95	72.95
32	Control group (300mg/day)	Male	62	20.8	A	No bacterial infection	13	4		27.66	Persistent viral activity	Alive	Alive	693	28.42	71.5
33	Intervention group (450mg/day)	Male	46		A	No bacterial infection	22	0	0.01073	127.93	Persistent viral activity	Alive	Alive	216	12.06	18.33
34	Control group (300mg/day)	Male	66		A	No bacterial infection	16	5	0.01203	32.27	Persistent viral activity	Alive	Dead**	1374	37.26	57.27
36	Control group (300mg/day)	Male	46	24.93	A	Bacterial infection	18	0			Viral clearance***	Alive	Alive	66.5	3.42	7.59
37	Control group (300mg/day)	Female	59	22.9	A	Bacterial infection	19	1	0.01522	23.98	Persistent viral activity	Dead	Dead	328.5	21.2	21.21
38	Intervention group (450mg/day)	Female	46	27.05	A	Bacterial infection	13	0	0.02762	93.21	Persistent viral activity	Alive	Alive	261	11.39	14.39
39	Control group (300mg/day)	Male	57	21.52	A	No bacterial infection	24	4	0.0161	133.06	Persistent viral activity	Alive	Alive	145.5	7.69	9.7
41	Control group (300mg/day)	Male	63	30	A	Bacterial infection	17	3	0.00677	97.2	Persistent viral activity	Alive	Alive	137	6.48	11.28
42	Intervention group (450mg/day)	Male	56	25.69	A	No bacterial infection	17	1	0.02558	25.42	Persistent viral activity	Alive	Alive	481	27.54	56.77
43	Intervention group (450mg/day)	Female	59	20.82	A	No bacterial infection	12	2	0.01043	80.83	Persistent viral activity	Alive	Alive	1026.42	43.27	44.27

Remarks:

Case 11 *The result of persistent viral activity was imputed to the patient who died before day 5 of recruitment to fulfil the intention-to-treat principle. Imputing the result of viral clearance to the early discharged patient and viral persistence to the early died patient.

Case 34 **This enrolled patient survived at 28 days but subsequently passed away in the hospital. The cause of death is unlikely related to the influenza infection or oseltamivir but possibly related to late nosocomial complications.

Case 36 ***The result of viral clearance was imputed to the patient discharged from ICU before day 5 of recruitment to fulfil the intention-to-treat principle.
